# Reaction of the carbonate Sibillini Mountains Basal aquifer (Central Italy) to the extensional 2016–2017 seismic sequence

**DOI:** 10.1038/s41598-022-26681-2

**Published:** 2022-12-27

**Authors:** Costanza Cambi, Francesco Mirabella, Marco Petitta, Francesca Banzato, Giulio Beddini, Carlo Cardellini, Davide Fronzi, Lucia Mastrorillo, Alberto Tazioli, Daniela Valigi

**Affiliations:** 1 Department of Physics and Geology, University of Perugia, Via Pascoli s.n.c., 06123 Perugia, Italy; 2 Department of Earth Sciences, Sapienza University of Rome, P.le A. Moro 5, 00185 Rome, Italy; 3 Istituto Nazionale di Geofisica e Vulcanologia, INGV, Sezione di Bologna, Via Donato Creti, 12, 40128 Bologna, Italy; 4 Department of Science and Matter Engineering, Environment and Urban Planning (SIMAU), Università Politecnica delle Marche, Via Brecce Bianche 12, 60131 Ancona, Italy; 5 Department of Sciences, Roma Tre University, Largo San Leonardo Murialdo 1, 00146 Rome, Italy

**Keywords:** Environmental sciences, Hydrology, Natural hazards, Solid Earth sciences

## Abstract

Hydrogeological perturbations in response to earthquakes are widely described worldwide. In carbonate aquifers, a post-seismic discharge increase is often attributed to an increase of bulk permeability due to co-seismic fracturing and the attention on the role of faults to explain the diversion of groundwater is increasing. We focus on the reaction of carbonate hydrogeological basins to extensional seismicity, taking as an example the effects of the Central Italy 2016–2017 seismic sequence, on the Basal aquifer of the Sibillini Mountains area. Geo-structural, seismological and ground deformation data were collected and merged with artificial tracer tests results and with a 4-years discharge and geochemical monitoring campaign. The main NNW-directed groundwater flow was diverted to the west and a discharge deficit was observed at the foot-wall of the activated fault system with a relevant discharge increase, accompanied by geochemical variations, at the fault system hanging-wall. The observed variations are consistent with the combined action of a permeability increase along the activated fault systems, which modified the predominant pre-seismic along-strike regional flow, and with hydraulic conductivity increase due to fracturing, determining a fast aquifers emptying. We show that the prevailing mechanism depends on the aquifer systems position with respect to the activated faults.

## Introduction

Earthquakes are capable to severely affect groundwater flow, determining discharge changes in both streamflow^1–6^ and springs^5,7–9^, along with modifications of groundwater geochemistry^10–12^.

Discharge increases after earthquakes can be explained by three main mechanisms^13^: (i) changes in static strain determining aquifer geometry modifications, pore pressure changes and variations of hydraulic gradient^4,14^; (ii) dynamic strain effects such as consolidation and shaking of water out of the unsaturated zone^1–3,15,16^; (iii) increased permeability due to dynamic strain^5,7,8,13,17^.

The attention on the role played by faults (extensional, contractional, strike-slip, etc.) on groundwater flow-paths is recently growing. It is known that faults can act frequently as drivers or barriers to groundwater flow, depending on geometry, kinematics and fault rock lithology^18–20^. Many authors focused their attention on the interaction between fault zones and groundwater flow-paths and developed physical models to determine how flow is affected by fault zones^21–25^.

Few recent works^26–28^ deal with the problem of the change of fault systems role in favouring or hampering groundwater flow after a seismic crisis^19,26,29^. Variations of spring and streamflow discharge after extensional earthquakes are common, for example, in the Italian Apennines^4,14,30–33^.

**Figure 1:**
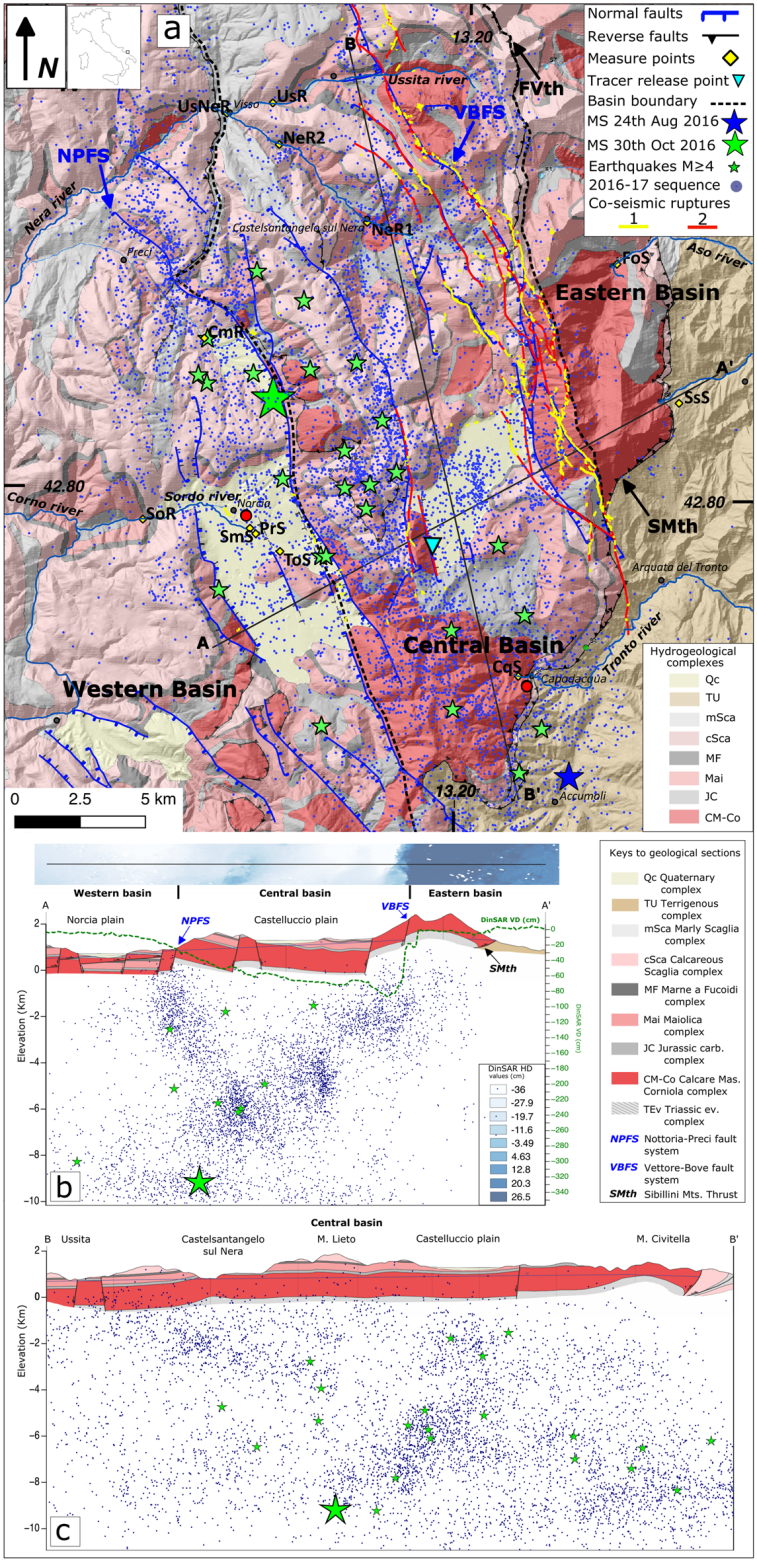
Integrated hydrogeological map and sections. The base map was obtained by elaborating the digital elevation model (DEM) from the TINITALY/01 DEM of the whole Italian territory (https://tinitaly.pi.ingv.it/; Tarquini et al.^41^; CC BY 4.0 License: http://creativecommons.org/licenses/by/4.0/), by means of the Open Source package QGIs v 3.16 (https://www.qgis.org/en/site/). The map has been then edited with the Open Source software Inkscape v.1.2.1 (https://inkscape.org/about/license/). (**a**) Hydrogeological map modified from^40^ (CC BY 4.0 License: http://creativecommons.org/licenses/by/4.0/) reporting also the main faults, Basins boundaries, distribution of earthquakes^35^ (CC BY 4.0 License: http://creativecommons.org/licenses/by/4.0/), co-seismic ruptures after: (1) Brozzetti et al. (ref^42^, Open access, Creative Commons Attribution 4.0 International License, 10.1029/2018tc005305); (2) Pucci et al. (ref^43^, free access available at 10.1002/2016GL071859; (3) Villani et al. (ref^44^, free access available at: 10.1029/2018TC005175), water discharge and geochemical measure points, tracer release and measure points. (**b**) Hydrogeological cross section with projection of earthquakes^35^ from a 4 km wide stripe. The green dashed line represents the vertical deformation (VD) recorded by DInSAR data^45,46^ (Open access Creative Commons CC BY license, 10.3390/rs10121901; 10.1038/s41598-022-07068-9). The bar above the cross section indicates the amount of horizontal deformation (HD) recorded by DInSAR data^45,46^. (**c**) Longitudinal hydrogeological section along the central basin with projection of earthquakes^35^ from a 7 km wide stripe showing that apart from surface co-seismic ruptures, earthquakes propagated up to shallow depths, where groundwater is hosted, in a widespread area. The discharge measure points (yellow diamond) acronyms are reported in Table 1.

The hydrogeological effects of the extensional 2016–2017 Central Italy seismic sequence (Mw_max_ = 6.5)^34,35^, involving the 600 km^2^ wide Sibillini Mountains area (Fig. 1a), were described by many authors^36,5,7–9,26,37^. A consistent increase of spring and stream discharge of the hydrogeological systems located at the hanging-wall of the activated SW-dipping normal fault system was observed.

These effects are attributed to an increase of bulk hydraulic conductivity, resulting in the increment of recession coefficients with respect to pre-seismic conditions^36,5,7–9^, to shaking and/or squeezing effects, or to breaching of hydraulic barriers^5,37^.

In this work we use the 2016–2017 seismic sequence as a paradigmatic example of the response of groundwater systems to extensional earthquakes. We highlight how, during and after extensional earthquakes, the response of springs and rivers to a seismic crisis depends on their position with respect to the activated faults.

We use geological, seismological and ground deformations data, integrated with discharge data coming from a 4-year monitoring period, to define how the geological framework and earthquakes-induced perturbations affect groundwater flow.

## Geological and tectonic setting

The Sibillini Mountains are part of the Umbria-Marche Apennines, an East-verging fold-and-thrust belt formed in the middle Miocene, later dissected by SW-NE directed extensional tectonics active since early Pleistocene, which formed quaternary continental basins and is still responsible for the present-day seismicity of the area^38,39^.

The reliefs of the area are formed by the Umbria Marche Stratigraphic Succession (UMSS, Early Jurassic-Oligocene), an over 2000 m thick calcareous multilayer, alternated with siliceous-marly and marly formations, overlying a Triassic evaporitic sequence (TEv). Three aquifer complexes can be identified^40^: the Calcare Massiccio-Corniola complex (CM-Co, Early Jurassic), the Maiolica complex (Mai, Early Cretaceous) and the Calcareous Scaglia complex (cSca, Cretaceous-Paleogene). At the regional scale CM-Co and Mai constitute the Basal aquifer^7^. The Jurassic carbonate complex (JC), the Marne a Fucoidi complex (MF, upper-early Cretaceous) and the Marly Scaglia complex (mSca, Eocene–Oligocene) act mainly as aquicludes.

The tectonic setting deeply influences groundwater flow. The Sibillini Mountains Thrust (SMth) is the main contractional tectonic structure and separates the carbonate Sibillini Mountains domain from the eastern terrigenous turbiditic deposits (Terrigenous Units complex, TU, Fig. 1a). The two main normal fault systems of the area are the NNW-SSE trending SW-dipping Vettore Bove Fault System (VBFS) and the Nottoria Preci Fault system (NPFS) and their antithetic segments (Fig. 1) which possess a cumulated stratigraphic throw of up to 1000 m each^42,47^.

The 2016–2017 sequence included more than 9 events with Mw > 5.0. The mainshocks occurred on August 24th (Mw = 6.0) and October 30th, 2016 (Mw = 6.5). Several co‐seismic surface ruptures were recorded after both mainshocks along the VBFS^42–44^.

## Results

We compared hydrogeological sections^40^ with earthquake locations^35^, and DInSAR data^45,46^ to build up an integrated geological model of the study area (Fig. 1).

We collected discharge data and analysed their evolution after the seismic crisis, comparing the results with pre-seismic conditions, focusing our attention on single and streambed springs fed by the Basal aquifer (CM-Co and Mai). The hydrogeological monitoring was coupled with a geochemical survey of groundwater. Post-seismic tracer tests were performed, and their results were compared with those of similar tests run before the seismic crisis.

### Geo-structural elaboration

The main contractional and extensional elements delimit three regional groundwater basins, hereafter named Eastern, Central and Western Basins (Fig. 1a), roughly corresponding to Basin 1, 2 and 3 proposed by Mastorillo et al.^7^

The Sibillini thrust (SMth) delimits eastward the Eastern Basin.

The Fiegni Vettore Thrust (FVth) separates the Eastern from the Central Basin to the north. More southward, the uplift of the TEv aquiclude up to an elevation higher than the saturated zone (Fig. 1b) acts as a lithological groundwater divide, located slightly East of VBFS^7,40^. Because of the northward axial plunging of the structure, the groundwater divide continues northward as a piezometric watershed^7^, separating the western and the eastern flow-paths, both characterized, in the pre-seismic, by a predominant NNW flow component sub-parallel to the main tectonic lineaments.

The Nottoria Preci Fault system (NPFS) can be generally considered as the hydrogeological limit between the Central and the Western Basins, although it was observed that prior to the seismic sequence groundwater coming from the Central Basin contributed to the feeding of Sordo river in the Norcia plain^5,7^. Groundwater flow in the investigated aquifer systems is difficult along E-W direction, while it is preferential along the strike of the main fault systems (SSE-NNW e.g.^26^).

The Eastern Basin represents the footwall of VBFS, while the Central one corresponds to its hanging wall. The Western Basin develops at the hanging-wall of the NPFS (Fig. 1b).

Despite of the presence of internal groundwater divides and of local groundwater flow exchanges between the Basins, the hydraulic separation among them is mainly operated by the fault systems, with the elevation of saturated zone progressively decreasing westward (Fig. 1b), as indicated by the elevation of punctual and streambed springs^40^ (Table S1).

During the 2016–2017 seismic sequence both VBFS and its eastward dipping antithetic fault were activated, as indicated by the hypocentres distribution^35^ (Fig. 1b).

Figure 1b,c show the earthquakes distribution along two perpendicular cross sections. Earthquakes occurred along the entire N-S elongation of the VBFS and propagated up to shallow depths, where groundwater is hosted, in a wide area also far from the sites where the co-seismic ruptures were mapped. Also events with Mw ≥ 4 were located at relatively shallow depths below the surface (Fig. 1c).

DInSAR data^45,46^ were sampled along section A–A′ in Fig. 1b and show that, in response to the seismic sequence, major co-seismic extension in the order of 20–25 cm and subsidence in the range of 90 cm was concentrated within the Central Basin and close to the VBFS. The activation of a fault antithetic to the VBFS favored co-seismic fracturing along with the Western and Central Basins boundary.

### Hydrogeological monitoring

**Figure 2:**
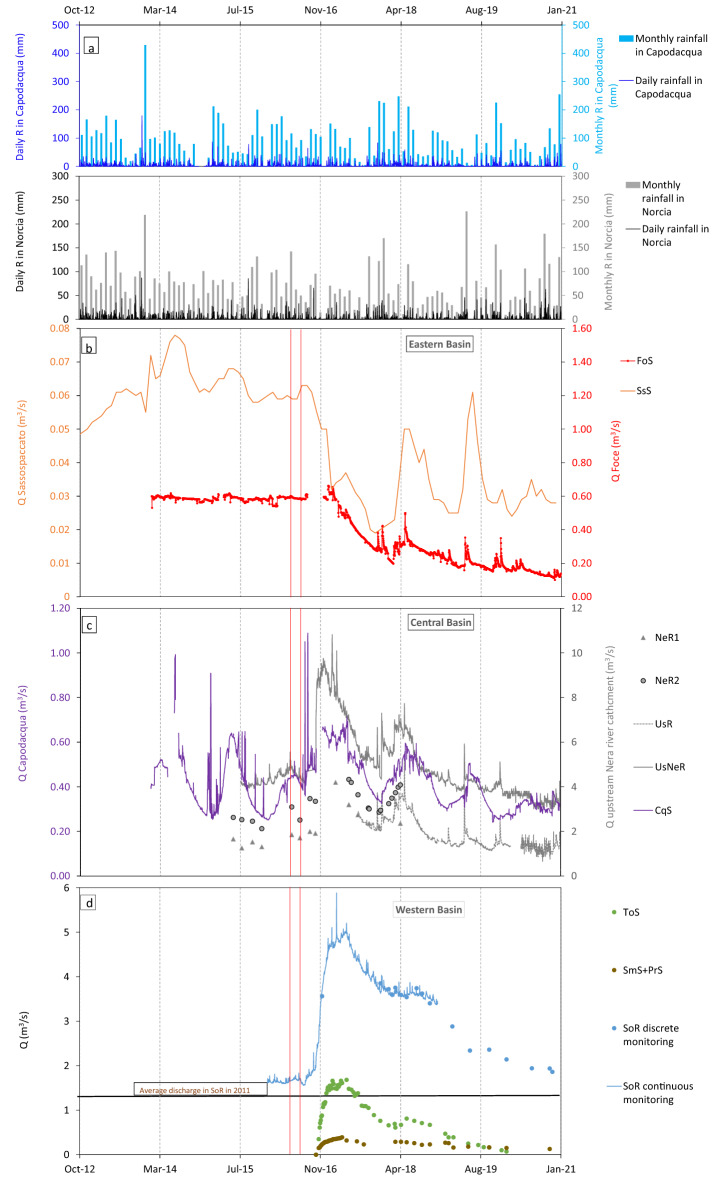
Daily rainfall in representative gauges and discharge of monitoring points. (**a**) Daily Rainfall (R) in Norcia and Capodacqua, respectively representative for systems located on the western and on the eastern slope. (**b**) Mean discharge (Q) of the main springs located in the Eastern Basin (VBFS footwall) at a daily (FoS) and monthly (SsS) scale. (**c**) Discharge (Q) of stream and rivers of the Central Basin (hanging wall of both VBFS and of its antithetic system). CqS is located on the Eastern slope while other measures (NeR1, NeR2, UsR, UsNeR) are taken on streams and rivers (streambed gaining springs) pertaining to the Upstream Nera River System, on the western slope. (**d**) Discharge (Q) of springs, streams and rivers of the Western Basin (hanging wall of VBFS and footwall of its antithetic). The red vertical bars indicate the main seismic shocks of August 24th 2016 (Mw = 6.0) and October 30th 2016 (Mw = 6.5). Location of the measures is reported in Fig. 1a.

Discharge data of the main springs and streams located in the Eastern, Central and Western Basins, recorded until December 2020, are shown in Fig. 2. The measurement sites are reported in Fig. 1a. Table 1 lists the measuring points shown in Fig. 1a, their acronyms, and position.

**Table 1:** List of acronyms used to indicate measuring points, location and brief description of each.

Name	Acronyms	Elevation (m a.s.l.)	Latitude	Longitude	Description
Foce Spring	FoS	944	42.88129	13.27134	Spring
Sassospaccato Spring	SsS	1300	42.83355	13.30186	Spring
Capodacqua Spring	CqS	841	42.73784	13.22894	Spring
Ussita River	UsR	649	42.93459	13.10763	Streambed spring along Ussita River
Nera River 1	NeR 1	729	42.89396	13.15332	Upstream streambed spring along Nera River
Nera River 2	NeR 1	641	42.92023	13.11107	Downstream streambed spring along Nera River
Nera River 3	UsNeR	612	42.93069	13.08617	streambed spring along Nera River downstream the merging with Ussita River
Torbidone Spring	ToS	617	42.77933	13.11573	Spring
San Martino Spring	SmS	600	42.78713	13.10137	Spring
Pratarella Spring	PrS	602	42.78527	13.10412	Spring
Sordo River	SoR	540	42.78944	13.05089	Streambed spring along Sordo River downstream the merging with San Martino and Pratarella Springs

Latitude and longitude are given in WGS 84 with an accuracy of about 1 m.

Discharge data were used to evaluate the effects of the seismic sequence in terms of variations of groundwater volume released by local and streambed springs before and after the seismic crisis. Concerning streambed gaining springs, volume variations were evaluated in the most downstream section of each investigated system, so that data from UsNeR and SoR were used.

After a careful analysis of the hydrographs, a common early post-seismic period (October 29th 2016–December 12th 2017), hereafter named early post-seismic, was chosen as a reference for all springs. A further post-seismic period, hereafter called overall post-seismic and including the investigated post-seismic time span (October 29th 2016–December 30th 2020) was also considered. Groundwater volume variations were quantified as the difference between the amount of groundwater effectively released by the springs in these intervals and the amount they would have released in the same time-spans if their pre-seismic average discharge had been maintained. Pre-seismic average was meant as the mean discharge of each time series shown in Fig. 2, from the first available value until October 28th 2016.

Table 2 shows the results of this analysis, which are comparable with those presented by Mastrorillo el al.^7^, obtained by using a slightly different data set and procedure.

**Table 2:** Variations of groundwater volume (ΔV) released by the main local and streambed springs in early and overall post-seismic reference periods. Volumes are measured in million cubic meters  (m^3^ × 10^6^) to make them immediately comparable wit each other.

Basin	Pre-seismic average discharge (m^3^/s)		ΔV early post-seismic (Oct. 29th 2016–Dec. 12th 2017) (m^3^ × 10^6^)		ΔV overall post-seismic (Oct. 29th 2016–Dec. 31st 2020) (m^3^ × 10^6^)	
Eastern	FoS (2014/01/07–2016/10/28)	SsS (2012/09–2016/10)	FoS	SsS	FoS	SsS
			− 5	− 1	− 42	− 4
	0.59	0.06	**− 6**		**− 46**	
Central	UsNeR (2015/08/17–2016/10/28)	CqS (2014/01/01–2016/10/28)	UsNeR	CqS	UsNeR	CqS
			+ 90	+ 4	+ 90	–
	4.41	0.41	** + 94**		+ 90^a^	
Western	SoR (2016/01/01–2016/10/28)		SoR		SoR	
	1.69		** + 91**		** + 246**	

Spring acronyms refer to Table 1 and to Fig. 1

Values in bold represent the overall variations of groundwater volume realesed by each Basin in the early and in the overall post-seismic periods.

^a^Underestimated value not inclusive of the CqS contribution.

The overall discharge of the Eastern Basin, represented by Foce spring (FoS) and to a lesser extent by the smaller Sassospaccato spring (SsS), decreased dramatically after the seismic crisis (Fig. 2b).

Since at least November 2016 FoS, the main outflow from Basal aquifer of the Eastern Basin, suffered a continuous negative discharge trend, which lowered the discharge down to 0.1 m^3^/s in late 2020 (Fig. 2b). A deficit of − 5 × 10^6^ m^3^ was estimated in FoS in the early post-seismic. During the overall post-seismic, the computed water deficit was − 42 × 10^6^ m^3^.

The same analysis on SsS shows that in the early post-seismic the water deficit of this spring was about − 1 × 10^6^ m^3^, while in the overall post-seismic it was about − 4 × 10^6^ m^3^ (Table 2).

The immediate reaction to the earthquake sequence of Capodacqua spring (CqS, 840 m a.s.l.), located on the south-east boundary of the Central Basin, is unknown, due to a gap in daily discharge measurements (Fig. 2c). Nonetheless, it can be observed that, although there were no significant rainfall events (Fig. 2a), in late 2016 CqS discharge was about 0.2 m^3^/s higher than before the mainshock (Fig. 2c). It was estimated that, in the early post-seismic, CqS released a water surplus of about 4 × 10^6^ m^3^. A negative trend in both maximum and minimum discharge was recorded from late 2017 to 2020 (Fig. 2c), although rainfall in this interval was not particularly low^8^. The negative trend forced the water company managing the spring, tapped for drinking purposes, to integrate its natural discharge by means of pumping wells. Therefore, it was not possible to evaluate, for overall post-seismic, the natural groundwater volume variation with respect to the pre-seismic.

Discrete monitoring data of the north-west area of the Central Basin (Figs. 1a and 2c) reveal that, after the seismic sequence, a significant discharge increase occurred in the streambed gaining springs of upstream Nera River catchment (sections NeR1, NeR2 and UsR, Fig. 1a). A relevant discharge increase also occurred downstream the confluence of the Ussita River in the Nera River (UsNeR section, Figs. 1a and 2c). The peak discharge in UsNeR was reached during January 2017. Between early 2017 and late 2019, despite a general negative trend of both minimum and maximum values, discharge in UsNeR, the most downstream section of the system, was higher or equal to pre-seismic values (Fig. 2c). The excess water drained by the Upstream Nera River system in the early post-seismic was as high as 90 × 10^6^ m^3^ (Table 2). The water surplus in the overall post-seismic was found to be still 90 × 10^6^ m^3^ (Table 2), indicating that the total amount of water surplus was released in the first year after the mainshocks.

A similar general discharge increase was observed in the Western Basin springs and streams, where the Torbidone Spring (ToS, Fig. 1a) was reactivated after the October 30th mainshock^9^. ToS, San Martino and Pratarella springs (SmS and PrS, Fig. 1a) and Sordo River (SoR, Fig. 1a) reached their discharge maximum in May 2017 (Fig. 2d), with a delay of about five months with respect to the Central Basin springs and rivers (Fig. 2c). Thereafter, all springs and rivers in Western Basin experienced a progressive discharge decrease, which led to the drying of ToS in summer 2020 while, despite of the gradual reduction, discharge of SoR was still higher than before the seismic crisis in December 2020 (Fig. 2d).

The discharge increase of ToS, SmS and PrS does not match the overall discharge excess of the downstream receiving SoR between December 2016 and November 2017 (Fig. 2d). SoR water surplus in the early post-seismic was about 91 × 10^6^ m^3^ (Table 2). A total amount of 45 × 10^6^ m^3^ was released in the same interval by ToS, SmS and PrS, which contribute for about a half to the total water excess released by SoR. This indicates that the discharge increase recorded in SoR is also due to an increase in the contribution of streambed gaining springs. In the overall post-seismic SoR released a water surplus as high as 246 × 10^6^ m^3^ (Table 2).

### Geochemical monitoring

**Figure 3:**
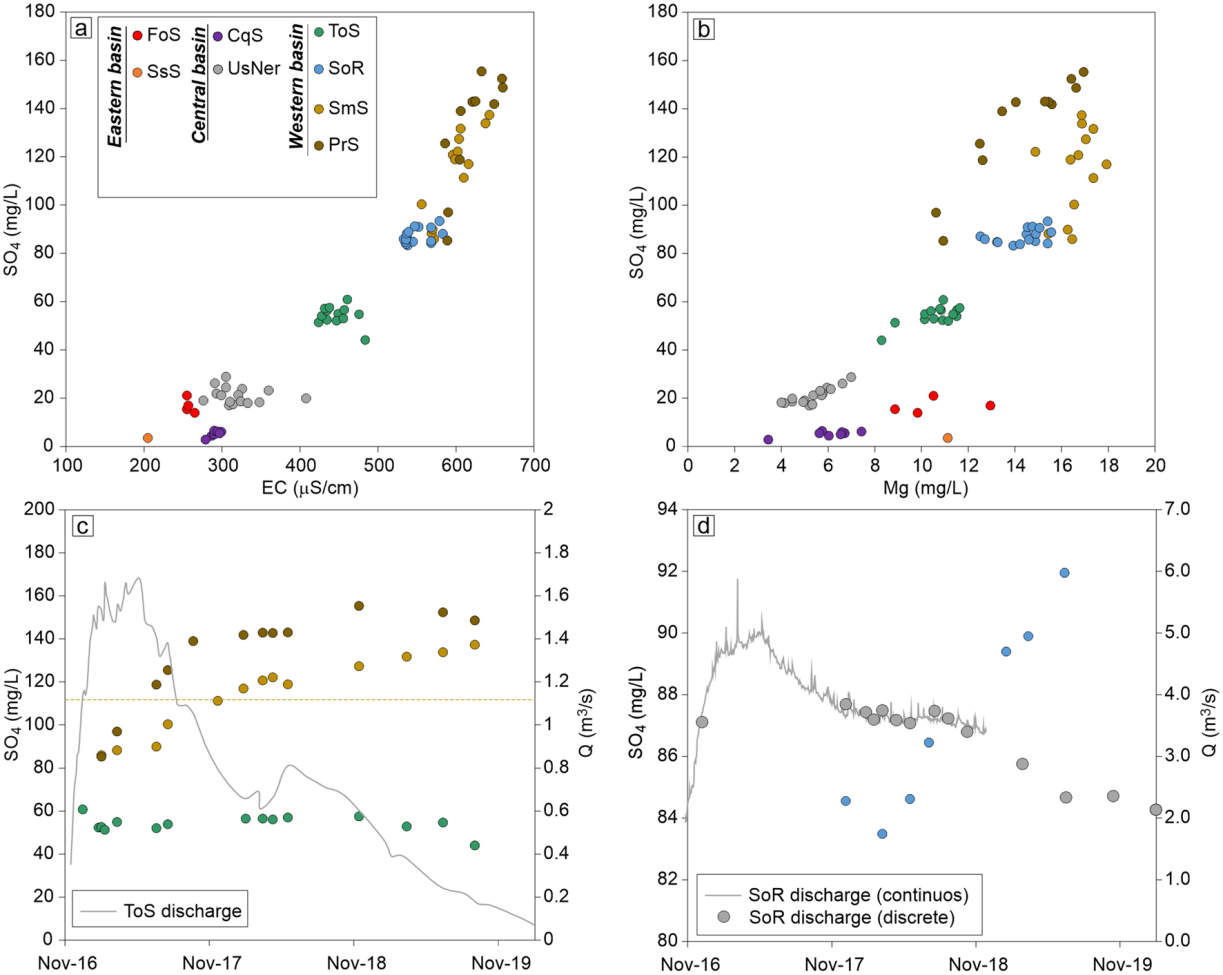
Geochemical features of sampled waters. (**a**) Electrical conductivity vs. SO_4_ concentration. (**b**) SO_4_ vs. Mg concentration. (**c**) Time variation of SO_4_ concentration of ToS, SmS and PrS compared with ToS discharges (grey line). The average SO_4_ concentration of SmS (January–February 2016) is also reported (dashed pale brown line). Symbols in all panels: Foce Spring (FoS, red dots), Sassospaccato Spring (SsS, orange dots), Capodacqua Spring (CqS, purple dots), Ussita-Nera River system (UsNeR grey dots), San Martino Spring (SmS, pale brown dots), Pratarella Spring (PrS, brown dots) Torbidone Spring (ToS, green dots) and Sordo River system (SoR, blue dots). UsNeR includes data from both springs and river waters upstream of UsNeR; SoR includes data from different location along Sordo river (Fig. S1).

All sampled spring and river waters (Fig. S1) show a Ca(Mg)-HCO_3_ composition typical of carbonate aquifers (Fig. S2). Nonetheless, Electric Conductivity (EC, a proxy of salinity), SO_4_ and Mg concentrations show a significant variability, from 205 µS/cm to 660 µS/cm, from ~ 2.7 to ~ 155 mg/L and from ~ 3.4 to ~ 19 mg/L respectively (Fig. 3). These variations occur both in space (Fig. 3a,b) and time (Fig. 3c,d), allowing to distinguish different water sub-types and their interactions. Higher salinity is generally associated to higher SO_4_ and Mg contents (Fig. 3a,b) and it can be related to a larger interaction with the dolomitic component of the CM-Co complex and with the evaporitic lithologies (TEv) stratigraphically below CM-Co (Fig. 1).

The main springs of the Eastern Basin (FoS and SsS) are characterized by a peculiar composition with relatively high Mg content, not associated to high SO_4_ concentration and salinity (Fig. 3a,b). This can be due to the relatively shallow and short groundwater flow-path to FoS and SsS, which are located at high elevation (910 m a.s.l. and 1130 m a.s.l. respectively).

The water compositions of the Central Basin highlight two water types with similar salinities but different SO_4_ contents and SO_4_/Mg ratios (Fig. 3a,b). Water discharged in the North-Western sector (i.e., UsNeR) has slightly higher salinity, higher SO_4_ contents and remarkably higher SO_4_/Mg ratio respect to those of the South-Eastern sector (i.e., CqS). These characteristics are reasonably due to longer circulation paths of the springs of North-Western sector, consistent with their lower elevation (~ 650 m a.s.l.) compared to CqS (~ 840 m a.s.l.) and to a lithological variability within the basin.

The Western Basin waters generally have the highest salinities, but also show different geochemical characteristics among each other. It is possible to distinguish two water types. The first one, hereafter named SMP hydrotype and including water from SmS and PrS, is more saline and SO_4_-rich respect to the other, hereafter named TOR hydrotype, which is represented by water emerging from ToS. TOR hydrotype has intermediate SO_4_, Mg and salinity between the SMP hydrotype and the groundwater of the Central Basin (Fig. 3a,b). Groundwater from SoR, sampled downstream of ToS, SmS and PrS, shows an intermediate composition between the two hydrotypes, with SO_4_ and Mg contents decreasing downstream.

Groundwater of SmS, PrS and SoR, showed the most evident variations following the 2016–2017 seismic sequence, with SO_4_ concentration that gradually increased since May 2017, while discharges of all springs in the Western Basin were simultaneously decreasing (Fig. 3c,d). On the contrary, these variations were not recorded in ToS, whose SO_4_ concentration remained practically steady (Fig. 3c) until the spring dried, in summer 2020. SmS gradually approached pre-seismic SO_4_ concentration (Fig. 3c) and SO_4_ content of both SmS and PrS became roughly stable in 2019. Conversely, the SoR positive trend in SO_4_ concentration continued until about half 2019 (Fig. 3d).

### Tracer tests

**Figure 4:**
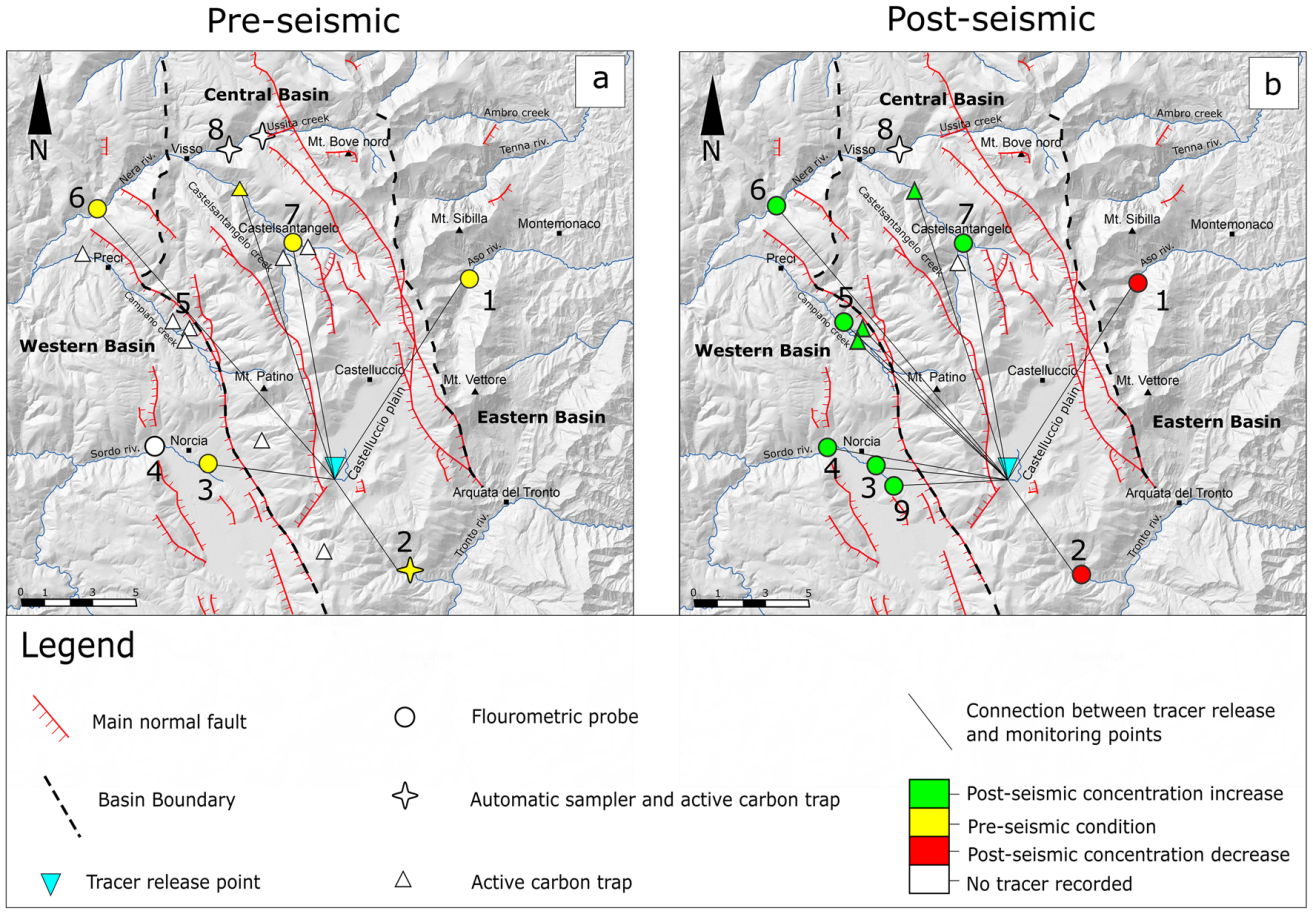
Tracer test results. The base map was obtained by elaborating the digital elevation model (DEM) from the TINITALY/01 DEM of the whole Italian territory (https://tinitaly.pi.ingv.it/; Tarquini et al.^41^; CC BY 4.0 License, http://creativecommons.org/licenses/by/4.0/), by means of the Open Source package QGIs v 3.16 (https://www.qgis.org/en/site/). The map has been then edited with the Open Source software Inkscape v.1.2.1 (https://inkscape.org/about/license/). (**a**) Distribution of tracer arrival at the sampling points before the seismic crisis. The yellow symbols indicate that tracer was detected. White symbols indicate that no tracer was detected. All numbered sampling points, but point 5, roughly correspond to water discharge measure points as follows: 1 = FoS; 2 = CqS; 3 = SmS/PrS; 4 ~ SoR; 6 downstream of UsNeR; 7 = NeR1; 8 = UsR; 9 = ToS. (**b**) Distribution of tracer arrival at the sampling points after the seismic crisis. The green symbols indicate that tracer was detected with increased concentration with respect to pre-seismic conditions. The red symbols indicate that tracer was detected with decreased concentration with respect to pre-seismic conditions. Connections between tracer release and monitoring point are not meant as actual pathways to the sampling sites but as an evidence of an existing link between the two.

Tracer tests were run during the pre- and post-seismic period^26,37,48^. Tracer was released in the Mergani sinkhole (Castelluccio plain, Figs. 1a and 4), located at an elevation of 1300 m a.s.l., about 350 m higher than FoS, the highest detection point (Table 1). This implies that the tracer movement involves the epiphreatic zone, characterised by fissure and karst patterns, before reaching the water table^49^. In the pre-seismic, groundwater traced flow was predominantly SSE-NNW oriented and mainly drained within the Central Basin, where the injection point is located (Fig. 4a). Only minor groundwater exchanges along East–West direction were observed with those from the Central to the Western Basin more relevant than those from the Central to the Eastern Basin.

After the seismic sequence, groundwater flow-paths significantly changed. The predominantly SSE-NNW oriented flow direction persists, but most of the tracer was recorded in the Western Basin springs, west of NPFS, also in springs and streams where the tracer was not detected before the seismic crisis (Fig. 4b). The hydraulic connection between Castelluccio plain and springs located southward within the Central Basin (CqS) is weakened in favour of a transfer from the injection point to springs and rivers located northward and westward. Tracer arrivals in the Eastern Basin decreased with respect to the pre-seismic conditions with only 0.9% of the total tracer amount collected in FoS. Specific rainfall events determine the arrival of the tracer to FoS in about ten days^48^, confirming that the main tracer movements towards the eastern basin involve the vadose passages of the epikarstic zone^49^. The transfer from the Central to the Eastern Basin is negligible with respect to the overall groundwater transfer among basins.

## Discussion

**Figure 5:**
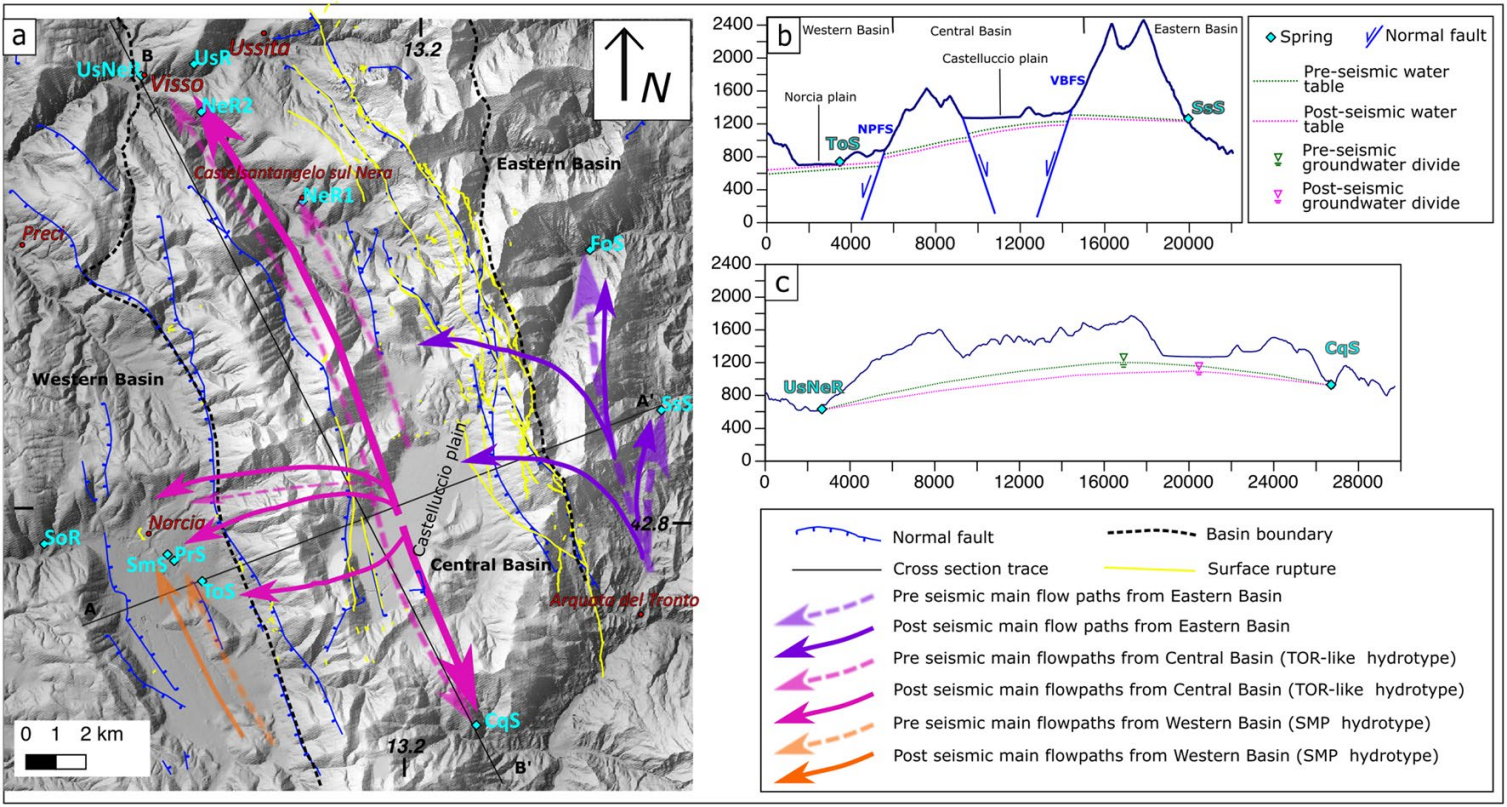
Conceptual scheme of hydrogeological modifications induced by the seismic sequence. The base map was obtained by elaborating the digital elevation model (DEM) from the TINITALY/01 DEM of the whole Italian territory (https://tinitaly.pi.ingv.it/; Tarquini et al.^41^; CC BY 4.0 License, http://creativecommons.org/licenses/by/4.0/), by means of the Open Source package QGis v 3.16 (https://www.qgis.org/en/site/). The map has been then edited with the Open Source software Inkscape v.1.2.1 (https://inkscape.org/about/license/). Surface ruptures data are from^42–44^ after: (1) Brozzetti et al. (ref^42^ Open access, Creative Commons Attribution 4.0 International License, https://doi.org/10.1029/2018tc005305); (2) Pucci et al. (ref^43^, free access available at https://doi.org/10.1002/2016GL071859; (3) Villani et al. (ref^44^ free access available at: https://doi.org/10.1029/2018TC005175). (**a**) Pre-seismic (dashed lines) and post-seismic (continuous lines) main flow-paths. Variations in line width represent increase or decrease of groundwater flow with respect to pre-seismic conditions. Different flow lines colours indicate different hydro-chemical characteristics. (**b**) Pre-seismic elevation of saturated zone in the three Basins. (**c**) Post-seismic modification of saturated zone elevation in Central Basin with potential migration of groundwater divide.

Normal faults mainly act as low permeability boundaries in the inter-seismic period, contributing to the separation of groundwater flow among the three Basins, with flowpaths mainly directed along the fault strike (SSE-NNW), despite some local transfers existing along the opposite direction (Fig. 5a). The elevation of saturated zone is highest in the Mt. Vettore Massif, at the foot-wall of VBFS, and progressively decreases westward (Figs. 1b and 5b).

The activation of VBFS and of its antithetic during the 2016–2017 seismic sequence determined a co-seismic fracturing of the fault zones^42–44^^.^. This induced an increase in hydraulic conductivity and a consequent westward migration of groundwater, previously stored in the Basal aquifer of Vettore Massif (Eastern Basin), towards the Central Basin, at the VBFS hanging-wall (Fig. 5a).

Such groundwater migration caused a lowering of the saturated zone within the Vettore Massif, which affected both FoS and SsS discharge, determining an overall water deficit in the Eastern basin of about − 6 × 10^6^ m^3^ in the early post-seismic and as high as − 46 × 10^6^ m^3^ in the overall post-seismic (Fig. 2b and Table 2). This last datum suggests that the westward directed groundwater migration through VBFS continued at least until the end of 2020, determining a progressively more severe groundwater deficit in the Eastern Basin.

The overall excess of groundwater released by the Central Basin is much higher than the water deficit of the Eastern Basin both in the in the early post-seismic (ΔV =  + 94.0 × 10^6^ m^3^) and in the overall post-seismic (ΔV =  + 90.0 × 10^6^ m^3^ not including the CqS contribution), as shown in Table 2. This suggests that a great portion of the discharge increase of the Central Basin was due to an overall increase of bulk permeability and to a process of fractures cleaning, resulting from the intense co-seismic fracturing. This caused a faster groundwater drainage, concentrated in the early post-seismic, as also testified by the post-seismic increase of spring depletion coefficients observed by other authors^36,8^ and by the increased tracer concentration recorded in the upper Nera River (point 7 in Fig. 4) after the seismic crisis.

The progressive aquifer emptying induced the lowering of the saturated zone within the Central Basin (Fig. 5c) and a subsequent gradual decrease in hydraulic gradient along the SSE-NNW flow direction, which determined the discharge lowering recorded since early 2017 (Fig. 2b). A drop of the saturated zone can influence the piezometric divide between UsNeR and CqS, also due to the additional drainage towards the western basin. The hypothesis of a widening of the aquifer sub-basin drained by UsNeR (Fig. 5c) and of a consequent restriction of CqS recharge area will require further investigations.

The increase of bulk permeability is congruent with seismological data, as the projection of earthquakes distribution along the geological sections (Fig. 1b,c) shows that shocks propagated up to the range of depths where groundwater is hosted, determining an increase of fracturing and hydraulic conductivity. Such increase is also consistent with DInSAR measurements^45,46^ showing that, after the 2016–2017 seismic sequence, major subsidence and extension, coherent with a significant permeability increase and a process of fractures cleaning, occurred at hanging-wall of VBFS, in the area corresponding to Central Basin (Fig. 1b).

In the Western Basin, the reactivation of ToS, the significant discharge increase in SmS and PrS, and the rise of SoR discharge were simultaneously observed after the seismic events (Fig. 2d). The evolution of hydro-chemical parameters, tracer test results and seismic data suggest that discharge increase in these systems was mainly due to an additional groundwater transfer from the Central Basin (Fig. 5a). Such transfer contributed further to the above-mentioned emptying of the Central Basin aquifer.

The activation of the fault system antithetic to VBFS determined an increase in hydraulic conductivity of fault systems, previously limiting the flow exchange towards the Western Basin. This interpretation is strengthened by the presence of surface co-seismic fracturing along both the fault antithetic to VBFS and NPFS (Fig. 5a).

Such interpretation is also supported by geochemical data. As resumed in Fig. 3c,d, since half 2017, a progressive increase of SO_4_ concentration, synchronous with the gradual discharge decrease of springs and rivers of the Western Basin, was observed in SmS, PrS and SoR, and not detected in ToS. This observation suggests that the initial discharge increase observed in these systems after the mainshock was mainly due to the arrival of a TOR-type groundwater component, similar to waters of the Central Basin and less saline than SMP (Fig. 2a,b). TOR was the only hydro-type emerging from the ToS, which was dry before the mainshock. Conversely, in SmS, PrS and SoR the TOR hydrotype was mixed with the previously emerging SMP hydrotype. The newly arrived TOR component determined a dilution of SMP in SmS, PrS and SoR, with a consequent shift of water hydrochemistry towards lower SO_4_ concentration. Since May 2017, TOR-type water gradually decreased, so discharge of all systems diminished, and SO_4_ concentration of SmS, PrS and SoR increased. It is worth to note that SO_4_ concentration of the SmS reached values similar to that of pre-seismic period (i.e., February 2016; Fig. 3c) in early 2018.

The SO_4_ concentration in SmS and PrS became roughly stable in late 2018, indicating that the influence of TOR-type water on the geochemical characteristics of these springs was negligible at that time. On the contrary the positive trend in SO_4_ concentration of SoR was still ongoing in June 2019 (Fig. 3d) while discharge was decreasing. This observation suggests that a progressively decreasing water component from the Central Basin (TOR like type) still contributed to the feeding of SoR streambed springs, located at lower elevation with respect to SmS and PrS.

Therefore, the increase of discharge observed in the Western Basin is mainly due to an additional water transfer from the Central one, favoured by the enhanced permeability of the fault zones, rather than to an increase of the overall hydraulic conductivity.

The hypothesis of increased permeability across the faut zones, inducing an additional water transfer from the Central Basin, is also supported by tracer tests results. As shown in Fig. 4, after the seismic sequence the amount of tracer reaching the Western Basin increased. The tracer was detected at points 4, 5 and 9, where it was not found before the earthquakes, and its amount increased at points 3 and 6.

The limited effect of hydraulic conductivity increase on the discharge rise of the Western Basin is congruent with the less intense vertical and lateral co-seismic deformations with respect to the Central Basin (Fig. 1b).

The different mechanisms responsible for the discharge increases observed in the Central and Western Basins explain the 5-month time shift in discharge peaks after the mainshock (Fig. 2c,d). The groundwater transfer from the Central to the Western Basin determined an increase of water table elevation, a consequent increase of hydraulic gradients and a post-seismic discharge excess lasting longer than in the Central Basin so that, in the overall post-seismic, the excess of groundwater volume drained by the Western basin has been as high as + 246 × 10^6^ m^3^ (Table 2).

In summary, two different mechanisms are considered responsible for the observed hydrogeological changes:

i. increase of permeability due to co-seismic rupturing along normal faults previously contributing to the separation of the Basins and suddenly activated during the seismic sequence. The rise of hydraulic conductivity along the fault zones was the relevant cause of an increase of the westward directed groundwater transfer, determining the overall and long lasting discharge increase in the Western Basin and of the water deficit recorded in the Eastern Basin (Fig. 5a).
ii. the increase of hydraulic conductivity of the Central Basin, due to intense co-seismic fracturing and fractures cleaning of the subsided and extended block between VBFS and its antithetic, which mobilized groundwater resources which would have otherwise been released in significantly longer times.

Groundwater flowed partly along pre-existing paths parallel to the fault strike (Central Basin Springs) and partly along paths perpendicular to the fault strike (Westward directed flow to the Central and Western Basins).

It can be assessed that, in case of extensional seismic sequences, the more relevant hydraulic permeability increase due to co-seismic fracturing must be expected at the hanging-wall of the activated fault, particularly when an antithetic fault system is activated, determining a relevant subsidence and consequent extension of the block located between the two fault systems.

The increase of permeability along the activated fault systems is also a key factor in modifying the groundwater transfer between hydrogeological basins mainly separated among each other by tectonic lineaments. Such transfer is driven by the pre-seismic piezometric distribution on both sides of the activated fault systems, with groundwater flowing from the higher to the lower saturated zone.

In the studied system, where two fault systems antithetic to each other were activated, the pre-seismic piezometric elevation coupled with the increase of the bulk permeability of the fault zones, determined the above-described westward groundwater transfer.

It is still unknown whether and when, after a transient-state condition period, the former equilibrium will be restored, or a new steady-state condition will be reached in the investigated area. The potential scenarios depend not only on the recovery of pre-seismic conditions in terms of bulk permeability of aquifers and fault role, but also on the groundwater recharge rate expected during next years to re-fill the emptied aquifers, which is largely uncertain, due to climate change threats.

This study stresses the importance of multidisciplinary studies for a better understanding of the reaction of complex carbonate fractured system to seismic sequences. It is shown how such reactions strongly depend on the location of aquifers with respect to geological structures. Understanding these mechanisms is of great importance as many carbonate aquifers, which contribute substantially to freshwater supply in many regions of the world and particularly in Southern Europe, are located in seismically active areas.

## Methods

### Elaboration of geological, seismic and DInSAR data

In order to understand the relationships between the subsurface geological setting and groundwater flow and how these may change across the seismic cycle we integrated different datasets, namely previous elaborated geological sections after^40^, earthquake locations after^35^ and DInSAR^45,46^. The geological sections are based on the hydrogeological map (Fig. 1a) which was validated with a set of both transversal and longitudinal sections to produce a consistent image of the subsurface. We choose two most representative geological sections, one transversal and one longitudinal, which, when compared with hydrogeological data, allow to infer the lithologies into which water is stored and flows towards both rivers and springs. Onto these sections we projected the distribution of earthquakes of the 2016–2017 seismic sequence after^35^, along a 4 km wide stripe. Data of the events with Mw ≥ 4 were extracted from the sequence itself to visualize the altimetric distribution of the moderate events and how they relate to the subsurface geology. Earthquake projection onto the geological sections was made using the tool “v.profile”, an add-on to the GRASS GIS package^50^. With the same procedure we plotted the distribution of both vertical and horizontal co-seismic deformation^45,46^. The results are shown in Fig. 1.

### Hydrogeological monitoring

The acquired data include discharge measurements of both local springs and stream sections. Discharges were measured by discrete and continuous monitoring by using flowmeters (FlowTracker by SonTek) and calibrated multiparametric probes (model by STS s.r.l.). The measures were integrated with data from the continuous monitoring of Umbria and Marche Regions (Servizio Rischio Idrogeologico, Idraulico e Sismico, Difesa del Suolo and SIRMIP, Protezione Civile Regione Marche). The frequency of measurements ranged from a minimum of one per week to a maximum of one per 15 min. Data were compared with those available for the pre-seismic period.

The location of all discharge measuring points is shown Fig. 1a while discharge time series are shown in Fig. 2.

### Geochemical sampling and analysis

The main groundwater discharge of the Sibillini Mountains Basal aquifer was sampled after the seismic crises, starting from the end of August 2016 up to September 2019, at both punctual springs and along the main rivers in correspondence of the main increases of their flow rate (i.e., at streambed springs).

Seventeen springs were sampled with a different frequency for a total of 96 samples. The spatial distribution of the sampling sites is shown in Fig. 1a. A larger spatial and temporal density of samples is available for the western sector of the basin. The complete dataset is reported in Supplementary material (Table S1).

PH, electrical conductivity (EC) and HCO_3_ were determined directly in the field. The HCO_3_ concentration was determined by acid titration with 0.01 N HCl using methyl orange as indicator.

Chemical analyses of major elements were performed by the Laboratory of Perugia University. Calcium and Mg concentrations were determined by atomic absorption (AA) flame spectroscopy on samples filtered through 0.45 μm membrane filters and then acidified directly in the field with 1% of 1:1 diluted HCl. Sodium and K were determined by atomic emission (AE) flame spectroscopy. AA and AE were performed using an Instrumentation Laboratory Spectrophotometer 951. Clorine and SO_4_ were determined by ion chromatography using a Dionex DX-120.

### Tracer tests

Several long time and periodic artificial tracer tests were conducted before, during, and after the seismic period in the Sibillini Mountains Area^26,48,49^.

The tracer release was performed by applying the sudden injection method in the Mèrgani sinking stream, located at an elevation of about 1300 m a.s.l. (Fig. 1a). The stream level was continuously measured by means of a hydrometric pressure transducer (CTD diver Eijkelkamp, accuracy ± 0.5 cmH_2_O and resolution 0.2 cmH_2_O) compensated by atmospheric pressure, using an acquisition time interval of 10 min, and the discharge was determined by a rating-curve.

Four tracer tests performed from February 2016 to June 2020, by using alternatively Na-Fluorescein (C_20_H_10_Na_2_O_5_) and Tinopal CBS-X (C_28_H_20_Na_2_O_6_S_2_), are of interest for this work. Technical details about these tests are reported in ^26,48,49^.

## Supplementary Information

Supplementary Figure S1.

Supplementary Figure S2.

Supplementary Table S1.

## Data Availability

Data generated or analysed during this study are included in this published article (and its Supplementary Information files) or are available from the corresponding author on reasonable request.
