# Supplementary figures and images for: Reaction of the carbonate Sibillini Mountains Basal aquifer (Central Italy) to the extensional 2016–2017 seismic sequence
Source: Sci Rep. 2022 Dec 27;12:22428. doi: 10.1038/s41598-022-26681-2 (PMC9794788; doi:10.1038/s41598-022-26681-2)

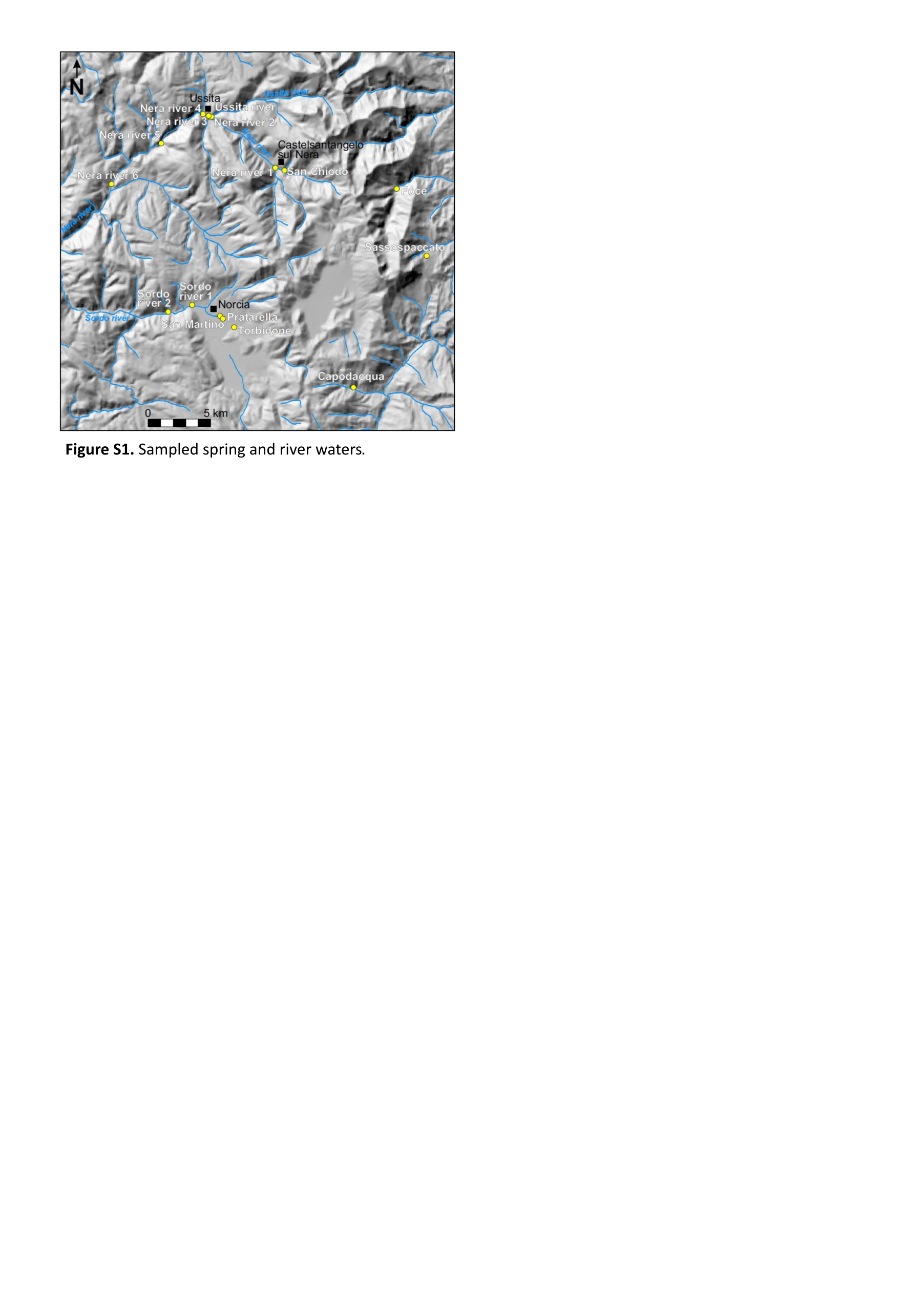

Supplement: Supplementary file 1 — Supplementary Figure S1. [file 41598_2022_26681_MOESM1_ESM.png]

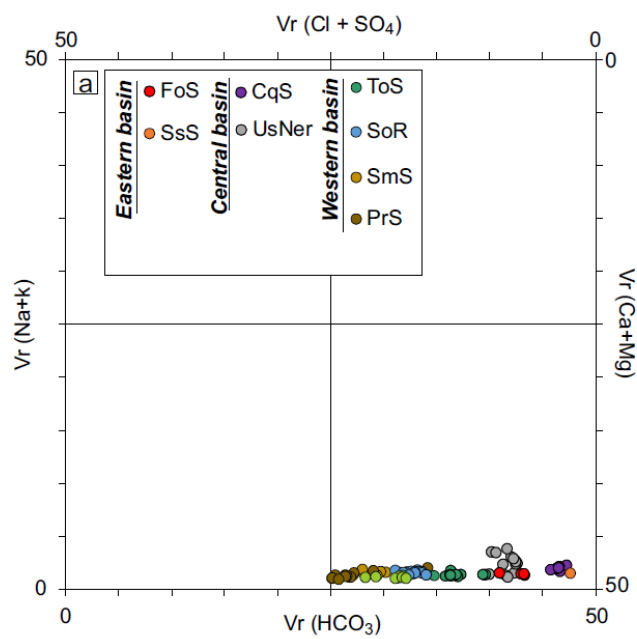

**Figure S2.** Composition of waters

Supplement: Supplementary file 2 — Supplementary Figure S2. [file 41598_2022_26681_MOESM2_ESM.pdf]
