# Supplementary material for: Reaction of the carbonate Sibillini Mountains Basal aquifer (Central Italy) to the extensional 2016–2017 seismic sequence
Source: Sci Rep. 2022 Dec 27;12:22428. doi: 10.1038/s41598-022-26681-2 (PMC9794788; doi:10.1038/s41598-022-26681-2)
Supplement: Supplementary file 3 — Supplementary Table S1. [file 41598_2022_26681_MOESM3_ESM.docx]

**Table S1 - Chemical and isotopic composition of sampled waters**

| **Sample**  **code** | **Name** | **Type** | **Data**  **(dd/mm/yyyy)** | **Latitude**  **(WGS84-UTM33)** | **Longitude**  **(WGS84-UTM33)** | **Elevation**  **(m a.s.l.)** | **T**  **(°C)** | **pH** | **EC**  **(μs/cm)** | **Ca**  **(mg/L)** | **Mg**  **(mg/L)** | **Na**  **(mg/L)** | **K**  **(mg/L)** | **HCO3**  **(mg/L)** | **Cl**  **(mg/L)** | **SO4**  **(mg/L)** | **δ^18^O**  **(‰ vs V-SMOW)** | **δD**  **(‰ vs V-SMOW)** | **References** |
| --- | --- | --- | --- | --- | --- | --- | --- | --- | --- | --- | --- | --- | --- | --- | --- | --- | --- | --- | --- |
| ToS | Torbidone | s | 16/12/2016 | 4738026 | 345834 | 641 | 9.6 | n.a. | 461 | 70.9 | 10.94 | 2.26 | 0.50 | 190.7 | 3.52 | 60.7 | -9.92 | -66.08 | This work |
| ToS | Torbidone | s | 25/01/2017 | 4738026 | 345834 | 641 | 9.7 | 7.54 | 435 | 68.2 | 10.91 | 2.48 | 0.39 | 192.9 | 3.41 | 52.3 | n.a. | n.a. | This work |
| ToS | Torbidone | s | 01/02/2017 | 4738026 | 345834 | 641 | 10.0 | 7.50 | 449 | 67.8 | 10.14 | 2.45 | 0.35 | 213.8 | 3.36 | 52.5 | -9.96 | -66.01 | This work |
| ToS | Torbidone | s | 10/02/2017 | 4738026 | 345834 | 641 | nd | 7.50 | 424 | 65.1 | 8.87 | 2.30 | 0.41 | 275.7 | 4.20 | 51.2 | -9.42 | -64.71 | This work |
| ToS | Torbidone | s | 13/03/2017 | 4738026 | 345834 | 641 | 9.9 | 7.56 | 449 | 69.5 | 10.15 | 2.19 | 0.50 | 200.7 | 3.28 | 54.8 | -9.83 | -66.13 | This work |
| ToS | Torbidone | s | 20/06/2017 | 4738026 | 345834 | 641 | 9.9 | 7.49 | 447 | 71.4 | 11.16 | 2.22 | 0.31 | 204.0 | 3.20 | 51.9 | -9.88 | -65.79 | This work |
| ToS | Torbidone | s | 19/07/2017 | 4738026 | 345834 | 641 | 10.0 | 7.75 | 428 | 68.7 | 11.52 | 2.33 | 0.42 | 204.4 | 3.20 | 53.8 | -9.81 | -65.60 | This work |
| ToS | Torbidone | s | 01/02/2018 | 4738026 | 345834 | 641 | 10.0 | 7.79 | 457 | 65.9 | 11.52 | 2.12 | 0.50 | 195.8 | 3.30 | 56.4 | -9.88 | -65.88 | This work |
| ToS | Torbidone | s | 16/03/2018 | 4738026 | 345834 | 641 | 10.1 | 7.57 | 434 | 69.2 | 10.84 | 2.45 | 0.60 | 205.6 | 3.50 | 56.4 | -10.16 | -66.66 | This work |
| ToS | Torbidone | s | 10/04/2018 | 4738026 | 345834 | 641 | 10.3 | 7.54 | 435 | 66.4 | 10.42 | 2.89 | 0.89 | 205.6 | 3.36 | 56.1 | -9.77 | -65.90 | This work |
| ToS | Torbidone | s | 18/05/2018 | 4738026 | 345834 | 641 | 9.9 | 7.57 | 432 | 66.5 | 10.80 | 2.11 | 0.99 | 207.7 | 3.48 | 57.0 | -9.44 | -63.23 | This work |
| ToS | Torbidone | s | 14/11/2018 | 4738026 | 345834 | 641 | 9.8 | 7.48 | 438 | 65.6 | 11.64 | 2.22 | 0.25 | 209.8 | 3.30 | 57.4 | -9.64 | -65.85 | This work |
| ToS | Torbidone * | s | 14/03/2019 | 4738026 | 345834 | 641 | 10.4 | 7.66 | 456 | 75.6 | 10.54 | 2.50 | 0.75 | 209.8 | 4.38 | 52.8 | -9.76 | -65.74 | This work |
| ToS | Torbidone * | s | 14/06/2019 | 4738026 | 345834 | 641 | 10.5 | 7.69 | 476 | 72.7 | 11.36 | 2.47 | 0.54 | 201.9 | 3.77 | 54.6 | -9.57 | -64.91 | This work |
| ToS | Torbidone * | s | 02/09/2019 | 4738026 | 345834 | 641 | 11.0 | 7.69 | 484 | 77.0 | 8.31 | 2.49 | 0.62 | 234.5 | 4.31 | 43.9 | -9.39 | -63.32 | This work |
| To2S | Torbidone 2 | s | 21/09/2017 | 4738026 | 345834 | 635 | 10.4 | 7.60 | 546 | 81.2 | 17.02 | 2.44 | 0.68 | 190.5 | 3.19 | 110.7 | -9.88 | -66.29 | This work |
| ToS2 | Torbidone 2 | s | 26/01/2018 | 4738026 | 345834 | 635 | 10.1 | 7.66 | 544 | 75.3 | 16.72 | 2.22 | 0.52 | 212.9 | 3.6 | 95.6 | -9.91 | -66.76 | This work |
| ToS2 | Torbidone 2 | s | 16/03/2018 | 4738026 | 345834 | 635 | 10.6 | 7.61 | 495 | 75.4 | 15.62 | 2.25 | 0.65 | 205.0 | 3.44 | 89.5 | -9.98 | -66.02 | This work |
| ToS2 | Torbidone 2 | s | 10/04/2018 | 4738026 | 345834 | 635 | 10.6 | 7.63 | 494 | 75.9 | 14.24 | 2.21 | 0.97 | 208.6 | 3.31 | 88.7 | -9.78 | -66.4 | This work |
| ToS2 | Torbidone 2 | s | 18/05/2018 | 4738026 | 345834 | 635 | 10.4 | 7.59 | 506 | 73.9 | 15.06 | 2.19 | 0.98 | 203.1 | 3.15 | 89.9 | -9.66 | -64.47 | This work |
| ToS2 | Torbidone 2 | s | 14/11/2018 | 4738026 | 345834 | 635 | 10.1 | 7.55 | 519 | 72.9 | 16.34 | 2.18 | 0.31 | 212.3 | 3.39 | 97.0 | -9.81 | -65.92 | This work |
| ToS2 | Torbidone 2* | s | 14/06/2019 | 4738026 | 345834 | 635 | 10.1 | 8.36 | 525 | 70.5 | 16.5 | 2.14 | 0.6 | 202.8 | 3.12 | 87.5 | -10.15 | -67.46 | This work |
| ToS2 | Torbidone 2* | s | 02/09/2019 | 4738026 | 345834 | 635 | 10.0 | 7.65 | 517 | 71.1 | 15.14 | 1.92 | 0.59 | 201.0 | 3.2 | 83.8 | -9.89 | -66.47 | This work |
| SmS | San Martino^#^ | s | 09/02/2016 | 4738924 | 344710 | 590 | n.a. | n.a. | n.a. | 90.4 | 19.09 | 2.86 | 0.79 | 220.3 | 5.32 | 115.5 | n.a. | n.a. | Data from UPM** |
| SmS | San Martino^#^ | s | 18/02/2016 | 4738924 | 344710 | 590 | n.a. | n.a. | n.a. | 88.7 | 18.73 | 2.81 | 0.68 | 219.4 | 5.53 | 116.8 | n.a. | n.a. | Data from UPM** |
| SmS | San Martino | s | 01/02/2017 | 4738924 | 344710 | 590 | 11.2 | 7.39 | 572 | 82.9 | 16.46 | 3.31 | 0.72 | 229.7 | 7.11 | 85.8 | -9.57 | -63.81 | This work |
| SmS | San Martino | s | 13/03/2017 | 4738924 | 344710 | 590 | 11.2 | 7.28 | 569 | 84.0 | 15.44 | 3.22 | 0.66 | 221.1 | 8.55 | 88.2 | -9.36 | -64.21 | This work |
| SmS | San Martino | s | 20/06/2017 | 4738924 | 344710 | 590 | 11.2 | 7.49 | 570 | 84.4 | 16.26 | 3.58 | 0.87 | 216.2 | 7.97 | 89.8 | -9.55 | -63.74 | This work |
| SmS | San Martino | s | 19/07/2017 | 4738924 | 344710 | 590 | 10.9 | 7.48 | 556 | 84.9 | 16.54 | 3.46 | 1.12 | 215.0 | 7.99 | 100.2 | -9.46 | -64.02 | This work |
| SmS | San Martino | s | 22/11/2017 | 4738924 | 344710 | 590 | 11.1 | 7.59 | 610 | 84.7 | 17.36 | 3.78 | 1.00 | 222.3 | 6.06 | 111.2 | -9.73 | -65.81 | This work |
| SmS | San Martino | s | 26/01/2018 | 4738924 | 344710 | 590 | 10.9 | 7.66 | 616 | 85.6 | 17.92 | 3.28 | 0.66 | 207.7 | 5.27 | 116.9 | -9.66 | -65.86 | This work |
| SmS | San Martino | s | 16/03/2018 | 4738924 | 344710 | 590 | 12.3 | 7.59 | 596 | 88.3 | 16.72 | 3.76 | 1.22 | 205.6 | 5.44 | 120.7 | -9.70 | -65.24 | This work |
| SmS | San Martino | s | 10/04/2018 | 4738924 | 344710 | 590 | 11.6 | 7.49 | 602 | 92.2 | 14.88 | 3.71 | 1.48 | 207.1 | 5.33 | 122.1 | -9.37 | -63.95 | This work |
| SmS | San Martino | s | 18/05/2018 | 4738924 | 344710 | 590 | 12.0 | 7.56 | 599 | 87.9 | 16.38 | 4.15 | 1.32 | 205.0 | 5.66 | 118.9 | -8.68 | -54.98 | This work |
| SmS | San Martino | s | 14/11/2018 | 4738924 | 344710 | 590 | 10.8 | 7.42 | 604 | 88.8 | 17.04 | 3.17 | 0.61 | 244.6 | 5.75 | 127.3 | -9.62 | -65.39 | This work |
| SmS | San Martino | s | 14/03/2019 | 4738924 | 344710 | 590 | 10.8 | 7.69 | 606 | 91.2 | 17.36 | 3.16 | 0.80 | 196.4 | 4.79 | 131.6 | -9.85 | -65.67 | This work |
| SmS | San Martino | s | 14/06/2019 | 4738924 | 344710 | 590 | 10.8 | 7.90 | 638 | 91.2 | 16.88 | 3.24 | 0.75 | 211.7 | 4.70 | 133.7 | -9.91 | -65.84 | This work |
| SmS | San Martino | s | 02/09/2019 | 4738924 | 344710 | 590 | 10.8 | 7.55 | 643 | 93.0 | 16.86 | 2.71 | 0.82 | 208.9 | 4.27 | 137.2 | -9.75 | -65.46 | This work |
| PrS | Pratarella | s | 01/02/2017 | 4738713 | 344931 | 590 | 11.7 | 7.30 | 589 | 92.7 | 10.94 | 3.55 | 2.78 | 248.3 | 4.03 | 85.2 | -9.72 | -64.63 | This work |
| PrS | Pratarella | s | 13/03/2017 | 4738713 | 344931 | 590 | 11.5 | 7.36 | 590 | 98.3 | 10.63 | 3.18 | 2.08 | 235.0 | 3.86 | 96.9 | -9.63 | -64.99 | This work |
| PrS | Pratarella | s | 20/06/2017 | 4738713 | 344931 | 590 | 12.2 | 7.32 | 605 | 95.0 | 12.62 | 3.15 | 2.57 | 216.2 | 3.23 | 118.7 | -9.96 | -66.86 | This work |
| PrS | Pratarella | s | 19/07/2017 | 4738713 | 344931 | 590 | 11.8 | 7.69 | 586 | 97.8 | 12.50 | 3.16 | 1.75 | 198.3 | 3.87 | 125.5 | -9.77 | -65.86 | This work |
| PrS | Pratarella | s | 21/09/2017 | 4738713 | 344931 | 590 | 11.2 | 7.50 | 606 | 96.7 | 13.46 | 2.87 | 1.27 | 188.3 | 3.26 | 138.9 | -9.86 | -65.85 | This work |
| PrS | Pratarella | s | 26/01/2018 | 4738713 | 344931 | 590 | 10.7 | 7.50 | 649 | 97.4 | 15.58 | 2.69 | 1.03 | 216.2 | 3.22 | 141.8 | -9.73 | -66.32 | This work |
| PrS | Pratarella | s | 16/03/2018 | 4738713 | 344931 | 590 | 10.9 | 7.45 | 623 | 100.3 | 15.44 | 2.95 | 1.30 | 208.3 | 3.42 | 142.8 | -9.91 | -66.23 | This work |
| PrS | Pratarella | s | 10/04/2018 | 4738713 | 344931 | 590 | 11.4 | 7.50 | 621 | 99.0 | 14.04 | 2.67 | 1.47 | 212.0 | 3.44 | 142.7 | -9.82 | -66.35 | This work |
| PrS | Pratarella | s | 18/05/2018 | 4738713 | 344931 | 590 | 12.5 | 7.53 | 625 | 100.0 | 15.28 | 2.59 | 1.52 | 209.5 | 3.38 | 142.9 | -9.49 | -64.4 | This work |
| PrS | Pratarella | s | 14/11/2018 | 4738713 | 344931 | 590 | 10.8 | 7.48 | 633 | 95.2 | 16.94 | 2.48 | 0.80 | 205.0 | 3.13 | 155.2 | -9.84 | -66.08 | This work |
| PrS | Pratarella | s | 14/06/2019 | 4738713 | 344931 | 590 | 10.9 | 7.80 | 659 | 95.2 | 16.42 | 2.47 | 0.92 | 201.9 | 3.38 | 152.3 | -10.02 | -66.19 | This work |
| PrS | Pratarella | s | 02/09/2019 | 4738713 | 344931 | 590 | 11.0 | 7.50 | 660 | 96.8 | 16.62 | 2.07 | 0.99 | 206.8 | 3.33 | 148.6 | -9.82 | -66.01 | This work |
| UsNeR | San Chiodo  Point 1^§^ | s | 06/09/2016 | 4750550 | 349858 | 795 | 8.7 | 7.95 | 305 | 48.5 | 6.99 | 1.46 | 0.70 | 153.7 | 2.49 | 28.7 | -10.38 | -68.53 | Fronzi et al., 2021 |
| UsNeR | San Chiodo  Point 1^§^ | s | 04/11/2016 | 4750550 | 349858 | 795 | 8.2 | 7.74 | 305 | 55.3 | 5.95 | 1.78 | 0.81 | 160.4 | 2.27 | 24.3 | -10.43 | -69.58 | Fronzi et al., 2021 |
| UsNeR | San Chiodo  Point 1^§^ | s | 20/04/2018 | 4750550 | 349858 | 795 | 8.3 | 8.00 | 291 | 45.2 | 6.62 | 1.66 | 0.32 | 151.7 | 2.88 | 26.0 | -10.08 | -67.87 | Fronzi et al., 2021 |
| UsNeR | San Chiodo  Point 3^§^ | s | 04/11/2016 | 4750550 | 349858 | 795 | 8.2 | 7.78 | 276 | 45.9 | 4.99 | 1.59 | 0.60 | 152.5 | 2.16 | 18.9 | -10.46 | -69.22 | Fronzi et al., 2021 |
| UsNeR | San Chiodo  Point 3^§^ | s | 28/11/2017 | 4750550 | 349858 | 795 | 7.9 | 7.74 | 293 | 44.5 | 5.77 | 1.39 | 0.41 | 158.1 | 2.52 | 21.8 | -10.55 | -70.54 | Fronzi et al., 2021 |
| UsNeR | Nera River | r | 20/07/2018 | 4750765 | 349122 | 725 | 9.1 | 8.27 | 299 | 46.6 | 5.73 | 1.69 | 5.16 | 161.3 | 7.29 | 21.1 | -10.49 | -70.02 | Fronzi et al., 2021 |
| UsNeR | Nera River | r | 26/06/2019 | 4750765 | 349122 | 725 | 10.7 | 8.63 | 326 | 48.0 | 6.12 | 1.27 | 0.47 | 173.2 | 2.51 | 23.7 | -10.28 | -68.87 | Fronzi et al., 2021 |
| UsNeR | Nera River | r | 20/07/2018 | 4754785 | 343960 | 620 | 11.1 | 8.14 | 309 | 52.4 | 5.20 | 1.84 | 4.35 | 177.2 | 7.00 | 16.9 | -9.86 | -65.51 | Fronzi et al., 2021 |
| UsNeR | Ussita River | r | 20/07/2018 | 4754843 | 344032 | 620 | 10.5 | 8.12 | 321 | 51.7 | 5.36 | 2.25 | 4.68 | 177.2 | 8.17 | 21.2 | -9.99 | -65.22 | Fronzi et al., 2021 |
| UsNeR | Ussita River | r | 26/06/2019 | 4754843 | 344032 | 620 | 12.9 | 8.51 | 360 | 52.5 | 5.67 | 3.01 | 0.55 | 176.0 | 4.59 | 23.1 | -9.76 | -64.45 | Fronzi et al., 2021 |
| UsNeR | Nera River | r | 20/07/2018 | 4754920 | 343799 | 615 | 11.0 | 8.01 | 314 | 51.7 | 5.33 | 1.84 | 2.75 | 186.1 | 5.94 | 17.4 | -10.29 | -68.82 | Fronzi et al., 2021 |
| UsNeR | Nera River | r | 20/07/2018 | 4755051 | 343351 | 605 | 8.0 | 11.80 | 310 | 51.9 | 4.94 | 2.10 | 2.07 | 181.5 | 5.15 | 18.4 | -10.02 | -67.49 | Fronzi et al., 2021 |
| UsNeR | Nera River | r | 20/07/2018 | 4752710 | 340009 | 504 | 12.6 | 8.28 | 324 | 55.1 | 4.46 | 2.79 | 1.89 | 188.8 | 5.78 | 18.5 | -10.02 | -66.52 | Fronzi et al., 2021 |
| UsNeR | Nera River | r | 26/06/2019 | 4752710 | 340009 | 504 | 14.6 | 8.60 | 408 | 56.8 | 4.47 | 2.75 | 1.88 | 188.2 | 4.83 | 19.8 | -9.99 | -64.53 | Fronzi et al., 2021 |
| UsNeR | Nera River | r | 20/07/2018 | 4749465 | 336035 | 456 | 12.2 | 8.14 | 333 | 56.5 | 4.13 | 3.07 | 2.03 | 184.2 | 6.43 | 17.9 | -9.85 | -65.64 | Fronzi et al., 2021 |
| UsNeR | Nera River | r | 26/06/2019 | 4749465 | 336035 | 456 | 13.7 | 9.02 | 348 | 58.2 | 4.01 | 2.90 | 5.07 | 192.2 | 8.96 | 18.1 | -9.89 | -64.83 | Fronzi et al., 2021 |
| SoR | Sordo River | r | 06/12/2017 | 4739807 | 342480 | 545 | 9.6 | 8.07 | 568 | 83.0 | 15.4 | 3.56 | 1.05 | 236.2 | 4.7 | 84.1 | -9.66 | -65.21 | This work |
| SoR | Sordo River | r | 09/03/2018 | 4739807 | 342480 | 545 | 12.1 | 8.11 | 538 | 84.7 | 13.94 | 3.47 | 1.17 | 215.3 | 5.16 | 83.2 | -9.44 | -64.38 | This work |
| SoR | Sordo River | r | 18/05/2018 | 4739807 | 342480 | 545 | 13.1 | 8.12 | 545 | 84.7 | 13.24 | 3.22 | 1.64 | 226.0 | 4.89 | 84.7 | -9.36 | -62.99 | This work |
| SoR | Sordo River | r | 05/07/2018 | 4739807 | 342480 | 545 | 13.2 | 7.93 | 537 | 82.7 | 12.52 | 3.22 | 1.86 | 238.5 | 5.8 | 87.1 | -9.9 | -65.91 | This work |
| SoR | Sordo River | r | 10/10/2018 | 4739807 | 342480 | 545 | 12.7 | 8.10 | 583 | 82.0 | 14.5 | 3.08 | 1.71 | 240.3 | 4.62 | 87.9 | -10.32 | -71.18 | This work |
| SoR | Sordo River | r | 17/01/2019 | 4739807 | 342480 | 545 | 6.5 | 8.21 | 552 | 82.2 | 14.54 | 2.97 | 1.27 | 222.4 | 4.79 | 90.8 | -9.55 | -64.78 | This work |
| SoR | Sordo River | r | 14/03/2019 | 4739807 | 342480 | 545 | 10.9 | 8.30 | 547 | 83.7 | 14.76 | 3.2 | 2.09 | 206.2 | 5.69 | 91.0 | -9.42 | -64.21 | This work |
| SoR | Sordo River | r | 14/06/2019 | 4739807 | 342480 | 545 | 14.7 | 8.35 | 579 | 84.7 | 15.4 | 3.06 | 1.56 | 221.7 | 4.52 | 93.3 | -9.81 | -65.36 | This work |
| SoR | Sordo River | r | 06/12/2017 | 4739260 | 340567 | 530 | 10.1 | 7.92 | 568 | 85.0 | 14.88 | 3.21 | 0.93 | 221.6 | 4.47 | 85.0 | -9.65 | -65.06 | This work |
| SoR | Sordo River | r | 09/03/2018 | 4739260 | 340567 | 530 | 13.4 | 7.96 | 536 | 84.1 | 14.22 | 3.25 | 1.02 | 234.9 | 4.79 | 83.7 | -9.39 | -64.25 | This work |
| SoR | Sordo River | r | 18/05/2018 | 4739260 | 340567 | 530 | 13.1 | 7.96 | 535 | 84.0 | 13.26 | 3.01 | 1.79 | 223.6 | 4.65 | 84.6 | -9.24 | -62.55 | This work |
| SoR | Sordo River | r | 05/07/2018 | 4739260 | 340567 | 530 | 12.4 | 7.48 | 533 | 80.1 | 12.72 | 3.04 | 0.87 | 226.9 | 4.76 | 85.8 | -9.71 | -65.37 | This work |
| SoR | Sordo River | r | 10/10/2018 | 4739260 | 340567 | 530 | 11.8 | 8.02 | 536 | 82.0 | 14.6 | 2.9 | 0.72 | 248.0 | 4.4 | 85.6 | -2.13 | -10.49 | This work |
| SoR | Sordo River | r | 17/01/2019 | 4739260 | 340567 | 530 | 10.1 | 8.01 | 537 | 83.4 | 14.9 | 2.82 | 0.96 | 254.4 | 4.47 | 88.0 | -9.59 | -64.64 | This work |
| SoR | Sordo River | r | 14/03/2019 | 4739260 | 340567 | 530 | 11.3 | 8.06 | 539 | 85.1 | 15.56 | 2.82 | 0.9 | 209.8 | 4.45 | 88.8 | -9.49 | -64.35 | This work |
| SoR | Sordo River | r | 14/06/2019 | 4739260 | 340567 | 530 | 13.6 | 8.18 | 568 | 82.8 | 15.06 | 2.77 | 0.89 | 226.6 | 4.62 | 90.6 | -9.53 | -63.95 | This work |
| FoS | Foce | s | 30/08/2016 | 4749082 | 358827 | 910 | 6.9 | 8.12 | 209 | 33.3 | 9.79 | 1.5 | 0.55 | 138.7 | 1.88 | 19.4 | n.a. | n.a. | This work |
| FoS | Foce | s | 07/10/2016 | 4749082 | 358827 | 910 | 6.8 | 8.07 | 258 | 33.4 | 9.76 | 1.15 | 1.05 | 136.6 | 1.93 | 19.8 | -10.37 | -68.16 | This work |
| FoS | Foce | s | 12/05/2017 | 4749082 | 358827 | 910 | 7.8 | 8.07 | 259 | 36.3 | 9.67 | 1.33 | 0.17 | 130.9 | 1.76 | 21.1 | -10.31 | -67.86 | This work |
| FoS | Foce | s | 15/09/2017 | 4749082 | 358827 | 910 | 6.6 | 8.09 | 255 | 33.6 | 10.52 | 1.75 | 0.09 | 132.7 | 1.62 | 21.0 | -10.25 | -67.39 | This work |
| FoS | Foce | s | 18/04/2018 | 4749082 | 358827 | 910 | 6.6 | 8.17 | 255 | 35.2 | 8.87 | 1.43 | 0.14 | 147.8 | 1.96 | 15.3 | -10.04 | -66.35 | This work |
| FoS | Foce | s | 02/10/2018 | 4749082 | 358827 | 910 | 6.6 | 8.15 | 257 | 37.8 | 12.96 | 1.59 | 0.65 | 154.3 | 2.21 | 16.8 | -10.12 | -67.49 | This work |
| FoS | Foce | s | 23/05/2019 | 4749082 | 358827 | 910 | 6.6 | 8.13 | 265 | 33.5 | 9.84 | 1.4 | 0.42 | 136.3 | 2.17 | 13.8 | -10.13 | -67.19 | This work |
| SsS | Sassospaccato | s | 30/08/2016 | 4743730 | 361213 | 1130 | 9.8 | 8.2 | 205 | 32.2 | 11.12 | 1.61 | 0.26 | 171.2 | 2.48 | 3.4 | -10.58 | -69.2 | This work |
| CqS | Capodacqua | s | 30/08/2016 | 4733216 | 355369 | 840 | 9.0 | 7.72 | 287 | 47.9 | 6.03 | 2.2 | 0.64 | 195.7 | 3.83 | 4.4 | -10.23 | -67.03 | This work |
| CqS | Capodacqua | s | 07/10/2016 | 4733216 | 355369 | 840 | 9.0 | 7.74 | 297 | 46.4 | 6.72 | 1.98 | 0.46 | 190.6 | 4.03 | 5.4 | -10.3 | -67.4 | This work |
| CqS | Capodacqua | s | 15/11/2016 | 4733216 | 355369 | 840 | 8.5 | 7.45 | 279 | 50.3 | 3.44 | 2.6 | 0.54 | 180.4 | 4.05 | 2.7 | -9.94 | -66.46 | This work |
| CqS | Capodacqua | s | 12/05/2017 | 4733216 | 355369 | 840 | 9.3 | 7.77 | 299 | 49.3 | 6.6 | 2.4 | 0.43 | 189.1 | 4.52 | 5.9 | -10.19 | -67.95 | This work |
| CqS | Capodacqua | s | 15/09/2017 | 4733216 | 355369 | 840 | 8.4 | 7.79 | 290 | 46.1 | 6.54 | 2.61 | 0.33 | 176.3 | 3.95 | 4.9 | -10.05 | -66.36 | This work |
| CqS | Capodacqua | s | 18/04/2018 | 4733216 | 355369 | 840 | 8.4 | 7.87 | 290 | 47.2 | 5.75 | 2.47 | 0.33 | 201.6 | 4.2 | 6.3 | -10.14 | -67.21 | This work |
| CqS | Capodacqua | s | 02/10/2018 | 4733216 | 355369 | 840 | 8.4 | 7.79 | 294 | 46.1 | 7.43 | 2.37 | 0.15 | 189.7 | 5.72 | 6.1 | -10.24 | -67.29 | This work |
| CqS | Capodacqua | s | 23/05/2019 | 4733216 | 355369 | 840 | 8.3 | 7.76 | 297 | 47.9 | 5.63 | 2.45 | 0.37 | 179.1 | 3.85 | 5.4 | -11.01 | -69.89 | This work |
